# Exploration of geographical population structure of *Anaplasma phagocytophilum*: Insights from 12 newly sequenced European human and bovine genome assemblies

**DOI:** 10.1016/j.crpvbd.2026.100393

**Published:** 2026-05-27

**Authors:** Clotilde Rouxel, Pierre Lucien Deshuillers, Meryl Vila-Nova, Deborah Merda, Pierre Boyer, Benoit Jaulhac, Claude Saegerman, Laurent Delooz, Grégoire Kuntz, Henri-Jean Boulouis, Nadia Haddad, Anne-Claire Lagrée, Virginie Chesnais

**Affiliations:** a ANSES, INRAE, Ecole Nationale Vétérinaire d’Alfort (EnvA), Laboratoire de Santé Animale, BIPAR, Maisons-Alfort, F-94700, France; b ANSES, Laboratoire de Sécurité Alimentaire, Laboratoire de Santé Animale, SPAAD, Maisons-Alfort, F-94700, France; c Université de Strasbourg, UR3073 PHAVI, CHRU de Strasbourg, Instituts de Bactériologie et de Parasitologie, 3, rue Koeberlé, Strasbourg, F-67000, France; d CNR des Borrelia, Hôpitaux Universitaires de Strasbourg, Strasbourg, F-67000, France; e Department of Infectious Diseases, Research Unit in Epidemiology, Risk Analysis and Biosecurity Applied to Veterinary Sciences, University of Liège, Liège, 4000, Belgium; f Regional Association for Animal Registration and Health (ARSIA) asbl, Ciney, 5590, Belgium; g Innoval, GDS Bretagne, Ploufragan, 22 440, France

**Keywords:** *Anaplasma phagocytophilum*, Short-reads targeted sequencing, Comparative genomics, Phylogenic analysis, Tick-borne fever (TBF), Human granulocytic anaplasmosis (HGA)

## Abstract

*Anaplasma phagocytophilum* is an obligate intracellular tick-borne bacterium widely distributed across the northern hemisphere. It is the causative agent of tick-borne fever in ruminants and human granulocytic anaplasmosis. The epidemiology of *A. phagocytophilum* infection differs markedly between the USA and Europe. In the USA, human granulocytic anaplasmosis represents an increasing public health concern, whereas infections in ruminants are rarely reported. In contrast, in Europe only sporadic human cases have been described, while infections in ruminants cause significant economic losses. Previous genomic studies based on limited datasets suggested genetic divergence between USA and European strains, but the small number of available genomes, particularly from Europe, limited robust conclusions. In this study, we report 12 new European *A. phagocytophilum* genomes, including four human-derived genomes and eight genomes from cattle. Using a capture-based short-read sequencing approach, we doubled the number of publicly available European genomes. In addition, systematic quality assessment of these public assemblies revealed variable genome completeness and the presence of contamination in several genomes. After curation of these datasets and inclusion of our 12 new sequenced genomes, comparative genomic analyses were performed using phylogenetic and population genetic approaches. Our results confirm a marked genetic divergence between European and USA strains. Hence, the capture-based short-read sequencing can be performed on samples from naturally infected humans and ruminants to increase the representation of European genomes, particularly human isolates, confirming *A. phagocytophilum* population structure patterns previously inferred from limited gene-based and provide new potential genomic markers that may help identify lineages associated with zoonotic transmission.

## Highlights

• Twelve new European *Anaplasma phagocytophilum* genome assemblies, including four new human-derived draft assemblies.
• Comparative genomics reveals strong population structure between USA/European and zoonotic/ruminant assemblies.
• Genome curation and expanded sampling identify potential markers associated with zoonotic lineages.

## Graphical abstract

**Figure:**
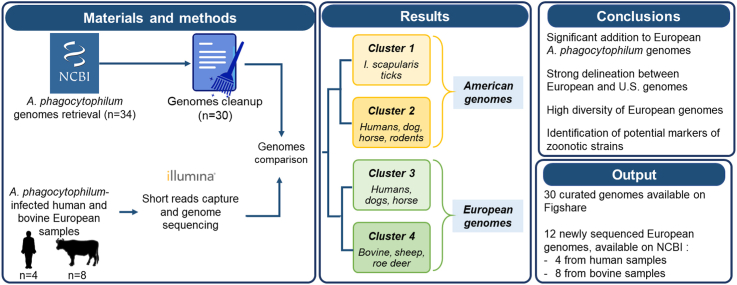

## 1 Introduction

*Anaplasma phagocytophilum*, the bacterium responsible for granulocytic anaplasmosis (GA), an emerging zoonotic disease with significant human and animal health consequences, is mainly transmitted by ticks belonging to the genus *Ixodes*, in particular *I. ricinus* in Europe, *I. scapularis* and *I. pacificus* in North America and *I. persulcatus* in Asia (Stuen et al., 2013). In the early 2000s, genetic analyses and similar biological and antigenic characteristics resulted in the merging of initially distinct bacteria from the genus *Ehrlichia* (*E. equi*, *E. phagocytophila* and the unnamed agent of human granulocytic ehrlichiosis) into a single bacterial species, *A. phagocytophilum* (Dumler et al., 2001), which is the causative agent of GA.

Several domestic and wild animal species may be infected with *A. phagocytophilum,* including cattle (*Bos taurus*), sheep (*Ovis aries*), horses (*Equus caballus*) and dogs (*Canis familiaris*). Clinical signs are most often nonspecific, such as fever, lethargy and anorexia. However, presentation and severity of clinical manifestations vary depending on the host and geographical location. Ruminant infection, known as tick-borne fever (TBF), is linked to decreased production and abortions, causing significant economic losses in Europe (Dugat et al., 2015). Conversely, no TBF case has been described in the USA to date.

The epidemiology of human *A. phagocytophilum* infection also differs greatly between the USA and Europe. In the USA, human granulocytic anaplasmosis (HGA) is an increasing health problem. In Europe, *A. phagocytophilum* is widespread in ticks and animals throughout the continent and average human seroprevalence is estimated at 8.3% (up to 31%) (Stuen et al., 2013; Matei et al., 2019; Azagi et al., 2020; Dixon et al., 2021; Rar et al., 2021). However, fewer than 300 HGA cases have been reported in Europe over the past years (Matei et al., 2019). Several hypotheses can explain this discrepancy. First, clinical cases may be underestimated, as several infections remain mild or asymptomatic. Seroprevalence may also be overestimated because of serological cross-reactivity (Matei et al., 2019; Dumler et al., 2005). Another possible explanation for the rarity of these cases in Europe could be the great genetic diversity and a host specialization of *A. phagocytophilum* (Jin et al., 2012; Rar et al., 2021), with only certain variants being zoonotic. For example, ruminant isolates are probably not zoonotic, whereas canine and equine isolates most likely are (Dugat et al., 2015). Additionally, wild reservoirs of European human and domestic animal isolates have yet to be identified, whereas in the USA, epidemiological cycles and reservoir hosts are well documented (Rar et al., 2021). Based on the 16S rRNA locus, two major variants, Ap-V1 and Ap-ha, can be distinguished in the USA. These variants can co-exist in the same geographical area and could be transmitted by the same vectors (Dugat et al., 2015). The white-tailed deer (*Odocoileus virginianus*) is a major reservoir host for Ap-V1, which is not considered zoonotic, but not for Ap-ha. In contrast, for the zoonotic variant Ap-ha, which is also pathogenic for dogs and horses, the main reservoir is the white-footed mouse (*Peromyscus leucopus*) (Dugat et al., 2015).

Studies based on *groEL* typing identified four ecotypes in Europe. Ecotype 1 has the widest range of hosts, and some of the genetic variants within this ecotype might be zoonotic (Jahfari et al., 2014; Jaarsma et al., 2019; Lesiczka et al., 2025). For example, strains belonging to Ecotype 1 have been detected in dogs, horses, wild boar (*Sus scrofa*), hedgehogs (*Erinaceus europaeus*), and red foxes (*Vulpes vulpes*). While these species could potentially act as reservoirs, further research is required to confirm this. Moreover, *groEL* typing, as well as other single genes, lack resolution to differentiate isolates associated with different clinical expressions in hosts (Lesiczka et al., 2025). Analysis of the complete genome following whole-genome sequencing is therefore essential to explore this question.

Despite the impact of anaplasmosis on human health in the USA, and its economic impact on ruminant herds in Europe, only 34 assembled genomes are currently available in the GenBank database. Among them, only five complete genomes (including 2 from dogs, 2 from sheep and one from a human Slovenian patient) are from Europe. This is due to the difficulty in sequencing new genomes. The difficulties associated with sequencing the genome of *A. phagocytophilum* are primarily due to the fact that it is an obligate intracellular bacterium located inside neutrophils. This complicates the isolation and culturing of the bacterium, which consequently makes further sequencing challenging, resulting in a lack of available genetic information. For example, the Slovenian patient isolate required culture in HL-60 cells prior to sequencing, which is time-consuming and difficult to achieve. Moreover, prior attempts by our group to sequence French bovine samples using Illumina® capture sequencing resulted in incomplete genomes and multiple scaffolds with host contamination, as it is difficult to separate the bacterial genome from that of the host (Dugat et al., 2014, 2016). Few studies have compared *A. phagocytophilum* genomes; a first pangenome study compared 28 *A. phagocytophilum* assemblies and found a clear separation between the USA and European genomes (Crosby et al., 2022). Lesiczka et al. (2025) recently compared 13 high-quality American and European genomes assembled into a single scaffold. They confirmed a strong delineation between isolates from the USA and Europe, and the close relation of the human Slovenian *A. phagocytophilum* genome with those derived from European dogs.

However, only a limited number of European genomes are currently available, including a single human-derived genome representative of clinical cases in Europe. To obtain a better representation of the genetic diversity in Europe, it is necessary to increase the number of *A. phagocytophilum* genomes obtained from both European human and animal samples. The first objective of this study was to evaluate the quality of the existing *A. phagocytophilum* genomes, and to propose a curated reference dataset by removing incomplete or contaminated genomes in order to minimize biases in subsequent genomic analyses. Then, we generated 12 new European *A. phagocytophilum* genomes obtained from four human clinical cases and eight domestic animal (bovine) samples, before performing comparative genomic analyses. This substantial expansion of the European genomic dataset confirms the strong separation between isolates from Europe and the USA, as well as between cattle and human isolates in Europe. Moreover, the significant increased number of European human genomes enables us to investigate new putative genetic markers associated with zoonotic strains.

## 2 Materials and methods

### 2.1 Public genome curation

In January 2026, 34 publicly available genomes of *A. phagocytophilum* were retrieved from the National Center for Biotechnology Information (NCBI) database. Genome quality was assessed to evaluate both contamination and completeness. Contamination status of genomes was evaluated using Kraken 2 (v 2.1.2) with minikraken2_v2_8 GB_201904_UPDATE database, by calculating the proportion of contigs not assigned to the genus *Anaplasma*. In order to reduce biases in downstream genomic analyses, contigs classified outside this genus were considered potential contaminants and were removed from the curated *A. phagocytophilum* database. Genome completeness and overall assembly quality were subsequently assessed using BUSCO (v.5.7.0) with the rickettsiales_odb10 database and QUAST (v.5.0.2) under default parameters. To retain informative genomes, assemblies with BUSCO scores <80% were excluded.

### 2.2 Collection of *A. phagocytophilum*-infected samples

DNA samples extracted from blood samples were obtained from four French human patients in 2016 and 2017 and collected at the National Reference Center for *Borrelia* in Strasbourg, France. Blood or endocervical swabs were collected from eight cows exhibiting signs of TBF from various locations in France and Belgium between 2013 and 2019. Blood samples from cattle were obtained by drawing blood from the caudal vein. Endocervical swabs were taken in the context of abortions. Blood from an aborted fetus was obtained in compliance with the protocol of Delooz et al. (2017).

### 2.3 DNA extraction and targeted sequencing

DNA was extracted from bovine blood samples and dried blood spots using the Nucleospin Tissue Kit (Macherey-Nagel, Düren, Germany). For the bovine blood samples, 25 μl of proteinase K, 200 μl of blood and 200 μl of Buffer B3 were mixed together and left to incubate at room temperature for 5 min. The samples were then lysed at 70 °C for 30 min at 800 rpm. DNA was then extracted according to the manufacturer’s instructions. The DNA was eluted with 40 μl of elution buffer. Dried blood spots on Whatman 3 MM CHRO blotting paper were cut into small pieces and placed in a 1.5-ml microcentrifuge tube. Then, 180 μl of Buffer T1 was added to the mixture, which was heated for 10 min at 94 °C. Next, 25 μl of proteinase K solution was added, followed by an incubation at 56 °C for 1 h. Finally, 200 μl of Buffer P3 was added, and the mixture was incubated at 56 °C for 10 min. The DNA was then extracted according to the manufacturer’s instructions and eluted in a final volume of 60 μl with elution buffer.

DNA extraction from the endocervical swabs was performed using the QIAamp™ DNA Mini Kit (Qiagen, Hilden, Germany) by Pas-de-Calais department analysis laboratory. After transferring the swab to 1 ml of PBS, 200 μl of the eluate was extracted and mixed with 180 μl of Buffer ATL and 20 μl of proteinase K, and the mixture was incubated for 30 min at 70 °C. Then, 200 μl of buffer AL was added, followed by a further incubation for 10 min at 70 °C. The DNA was then extracted according to the manufacturer’s instructions and eluted with 200 μl of elution buffer. All samples were stored at −20 °C.

Human samples were extracted using the QIAcube device (Qiagen, Venlo, The Netherlands) according to the manufacturer’s instructions, with the Cador Pathogen QIAcube HT Kit.

For each sample, the DNA quality was evaluated spectrophotometrically using absorbance ratios of 260/280 nm and 260/230 nm (Nanodrop, Thermo Scientific, Waltham, USA). The concentration of the DNA was determined using a Qubit 2.0 fluorometer with the Qubit DNA BR assay kit (ThermoFisher Scientific, Waltham, USA).

All bovine DNA samples were tested for the presence of *A. phagocytophilum* DNA with a Taqman real-time PCR targeting a 77-bp region of the gene encoding the major surface protein 2 (*msp2*) (Courtney et al., 2004). qPCR was performed with 2 μl of DNA according to the protocol of Rouxel et al. (2024) (Ct values are indicated in Supplementary Table S1).

Human DNA samples were tested using a Taqman real-time PCR applied to amplify a 73-bp fragment from the *A. phagocytophilum msp2/p44* gene (Koebel et al., 2012).

Targeted sequencing was performed by Helixio (Saint Beauzire, France) using a SureSelect design including 56,116 custom probes. The probes were designed based on seven reference genomes, available at the scaffold level (6/7) or at the contig level (1/7) and are available on Figshare repository (https://doi.org/10.6084/m9.figshare.32083359). These reference genomes were selected in order to be representative of geographical and host diversity (NCBI GenBank accessions GCA_000013125.1, GCA_000964745.1, GCA_000964785.1, GCA_900000025.1, GCA_000439795.1, GCA_013487825.1, and GCA_000964685.1). Paired-end sequencing of the libraries was performed using 500 ng of input DNA on an Illumina® NextSeq 500 platform, generating 2 × 76 bp reads according to the manufacturer’s instructions.

### 2.4 Checking for cross-contamination in sequencing results

*In silico* mixed-infected samples (*n* = 51) were generated by mixing sequencing reads from real samples. Contamination ratios were randomly selected between 10% and 40% to simulate varying levels of contamination. Subclonal mutations were identified by mapping reads to the reference genome GCA_002849375.1 using Minimap2 (v.2.24-r1122) (Li, 2021), followed by variant calling using LoFreq (v.2.1.5) (Wilm et al., 2012). Allele frequencies (AF) were extracted from the final VCF files, and a comparison was performed between *in silico* mixed-infected samples and the real sample.

### 2.5 Assembly of sequenced isolates and comparative genomic analysis

Raw sequencing reads were quality-filtered and trimmed using fastp (v.0.22.0) (Chen et al., 2018) to remove low-quality bases, adapter and primers sequences. Host-derived sequenced reads were filtered by alignment against the curated *A. phagocytophilum* database using Minimap2 (v.2.24-r1122) (Li, 2021), and non-matching reads were discarded. The remaining reads were assembled *de novo* using Shovill (v.1.1.0) (https://github.com/tseemann/shovill).

To compare genomic content among isolates sequenced in this project and genomes from public databases, average nucleotide identity (ANI) was calculated using PyANI (v.0.4.1) (Pritchard et al., 2015) with the *average_nucleotide_identity.py* script. Mash (v.2.3) (Ondov et al., 2019), a k-mer method that uses hash sketching was also applied to rapidly estimate pairwise genomic distances between sequences.

Phylogeny was performed after core genome single-nucleotide polymorphisms (core-SNPs) identification using snippy (v.4.6.0) (https://github.com/tseemann/snippy) with GCA_002849375.1 as a reference for read mapping. Differences in the number of SNPs between populations were assessed using a Mann-Whitney test using python (v.3.11) and scikit-learn (v.1.7.2), as SNP counts did not meet the assumptions of normality. A maximum-likelihood phylogenetic tree was reconstructed from core-SNP alignment using IQ-TREE (v.3.0.1) (Wong et al., 2026). The best-fitting nucleotide substitution model was selected automatically, and branch support was assessed using standard bootstrap approaches. Population structure was inferred using FastBAPS (v.1.0.8) (Tonkin-Hill et al., 2019), a Bayesian hierarchical clustering approach applied to the core-SNP alignment. Recombination was inferred using ClonalFrameML (v.1.12) (Didelot and Wilson, 2015) from the core genome alignment and corresponding maximum-likelihood phylogeny. The relative contribution of recombination to genetic diversification (r/m) was calculated as (R/θ) × δ × ν, where R/θ is the rate of recombination relative to mutation, δ is the mean recombination tract length, and ν is the probability that a nucleotide in the recombined fragment differs from the recipient genome. Genetic differentiation between populations was quantified using pairwise *F*_ST_ (fixation index: a.k.a. differentiation index) statistics with R packages *adegenet* (v.2.1.10) and *hierfstat* (v.0.5.11).

### 2.6 Genome-wide association analysis

Pangenome analyses were performed directly on genome assemblies in FASTA format using PPanGGOLiN (v.2.1.2) (Gautreau et al., 2020). The core genome was defined as the set of genes found in more than 95% of isolates, while less represented genes were grouped into the accessory genome. The genes’ presence-absence matrix served as input for Genome-Wide Association Studies (GWAS), which were conducted with PySEER (v.1.3.11) (Lees et al., 2018). To account for population structure, GWAS used a phylogeny-based distance matrix as a covariate. Comparisons between human-derived isolates and isolates from other sources were performed to identify potential zoonotic markers.

## 3 Results

### 3.1 Public genomes database curation

As of January 2026, 34 *A. phagocytophilum* references were publicly available in NCBI databases, ranging from complete assemblies to draft contigs. The assemblies contained variable proportions of contigs assigned to the genus *Anaplasma* (42–100%), indicating substantial host contamination in some cases. The assemblies were therefore curated by removing contigs not assigned to the genus *Anaplasma*, after which BUSCO completeness ranged from 79% to 98%. Excluding assemblies with BUSCO scores < 80% yielded a dataset of 30 *A. phagocytophilum* genomes of sufficient completeness. The final dataset comprised genomes ranging from complete scaffolds to contig-level assemblies (1–299 contigs). Genome sizes ranged from 1.26 to 1.79 Mb, and BUSCO completeness scores ranged from 84.6% to 98.1%. The final list of the cured *A. phagocytophilum* genome dataset is provided in Table 1.

**Table 1:** List of the final curated dataset of 30 *A. phagocytophilum* genomes, including host, country of origin and collection year, as well as sequencing quality scores and assembly data.

Accession	Strain name	Host	Country	Sequencing	Length (bp)	No. of contigs	N50	BUSCO score
GCA_000013125.1	HZ	*Homo sapiens*	USA (NY)	NA	1,471,282	1	1,471,282	98.1
GCA_000439755.1	HZ2	*Homo sapiens*	USA (NY)	454	1,477,581	1	1,477,581	98.1
GCA_000968465.1	HGE1 mutant	*Homo sapiens*	USA (MN)	PacBio	1,493,666	4	1,124,244	97.6
GCA_002849375.1	NY18	*Homo sapiens*	USA (NY)	Illumina	1,387,508	142	17,308	94.0
GCA_000478425.1	HGE1	*Homo sapiens*	USA (MN)	454	1,469,600	2	1,088,218	98.1
GCA_000964685.1	Webster	*Homo sapiens*	USA (WI)	PacBio	1,479,407	1	1,479,407	97.8
GCA_000964725.1	NCH-1	*Homo sapiens*	USA (MA)	PacBio	1,502,749	15	185,182	95.1
GCA_000964935.1	HGE2	*Homo sapiens*	USA (MN)	PacBio	1,482,055	1	1,482,055	97.8
GCA_000964945.1	ApWI1	*Homo sapiens*	USA (WI)	PacBio	1,497,074	1	1,497,074	98.1
GCA_000964985.1	ApNYW	*Homo sapiens*	USA (NY)	PacBio	1,503,088	16	221,185	96.7
GCA_000439795.1	Dog2	*Canis familiaris*	USA (MN)	454	1,473,302	1	1,473,302	98.1
GCA_000965125.1	Annie	*Equus caballus*	USA (CA)	PacBio	1,510,628	14	250,970	96.7
GCA_000689655.1	MRK	*Equus caballus*	USA (CA)	454	1,479,231	9	831,519	98.1
GCA_000439775.1	JM	*Zapus hudsonius*	USA (MN)	454	1,481,598	1	1,481,598	98.1
GCA_000968455.1	CR1007	*Tamia sibiricus*	USA (MN)	PacBio	1,499,008	3	1,323,113	91.8
GCA_000478445.1	CRT38	*Ixodes scapularis*	USA (MN)	454	1,506,545	2	1,125,951	97.8
GCA_000689615.1	CRT35	*Ixodes scapularis*	USA (MN)	454	1,447,016	25	204,176	98.1
GCA_000964915.1	CRT53-1	*Ixodes scapularis*	USA (MN)	PacBio	1,553,070	43	61,606	94.5
GCA_000964745.1	ApMUC09	*Canis familiaris*	Netherlands	PacBio	1,520,397	1	1,520,397	89.8
GCA_000964785.1	ApNP	*Canis familiaris*	Austria	PacBio	1,521,576	1	1,521,576	84.6
GCA_050955495.1	SLO-1	*Homo sapiens*	Slovenia	ONT; Illumina	1,517,188	1	1,517,188	98.1
GCA_013487825.1	Norway v1	*Ovis aries*	Norway	PacBio; Illumina	1,564,418	1	1,564,418	97.6
GCA_000689635.2	Norway v2	*Ovis aries*	Norway	454; PacBio	1,545,197	1	1,545,197	98.1
GCA_900000025.1	BOV-10_179	*Bos taurus*	France	Illumina	1,370,818	169	14,719	94.2
GCA_900078505.1	C1	*Bos taurus*	France	Illumina	1,600,863	198	19,152	95.6
GCA_900088625.1	C3	*Bos taurus*	France	Illumina	1,492,967	147	18,865	95.4
GCA_900088645.2	RD1	*Capreolus capreolus*	Germany	Illumina	1,515,203	274	6989	91.5
GCA_900088655.2	H2	*Equus caballus*	France	Illumina	1,766,702	299	7676	89.0
GCA_023278635.1	KZ-A1	*Homo sapiens*	South Korea	PacBio	1,449,336	1	1,449,336	97.8
GCA_023476575.1	AKS2020-120P	*Marmota himalayana*	China	Illumina	1,250,011	185	10,585	95.9

*Abbreviation*: NA, not available.

The public genomic dataset was predominantly composed of American samples (18/30, 60%), particularly human *A. phagocytophilum* genomes from the USA (10/30, 33%). European samples were less represented, with 10 out of 30 genomes (33%), including only a single human *A. phagocytophilum* genome from Slovenia. To address this gap, we sequenced 12 new samples obtained from both bovine and human hosts.

### 3.2 New genome draft assemblies of *A. phagocytophilum*

We first confirmed the absence of detectable mixed-infection of *A. phagocytophilum* in our samples by assessing subclonal mutations. Variant calling results were compared to *in silico* datasets artificially mixed-infected. Our samples showed far fewer SNPs than contaminated controls (452 *vs* 7653). Allele frequency distributions were also different. Mixed-infected *in silico* datasets showed a median allele frequency (AF) of ∼0.9, with many variants below 0.7, consistent with the simulated contamination levels, whereas our sequenced samples displayed a median AF of 0.99, indicative of predominantly clonal infections (Supplementary Fig. S1).

Assemblies from targeted sequencing were generated using reads specific to the genus *Anaplasma*, resulting in shorter assemblies than those previously reported, with a median assembly length of 1.3 Mb. As expected for targeted sequencing approaches, assemblies were relatively fragmented, with a median of 255 contigs. This fragmentation was in line with other public genomes obtained with Illumina® (Table 1). However, the GC content of the assemblies was consistent with expectations (i.e. a median of 41%), and BUSCO scores remained high (90–95%), indicating levels of completeness comparable to those of publicly available genomes, and suggesting a limited impact of potential methodological biases on genome completeness (Table 2). Overall, these additions more than double the number of available European *A. phagocytophilum* genome draft assemblies.

**Table 2:** List of the 12 newly sequenced *A. phagocytophilum* genome draft assemblies, including the host, their country of origin and collection year, as well as sequencing quality scores and assembly data.

Isolate ID	Host	Country (department)	Year	Sample type	Length (bp)	No. of contigs	BUSCO score
FRE-Bta-1	Bovine	France (62)	2019	Blood	1,273,448	283	93.4
FRE-Bta-2	Bovine	France (56)	2013	Blood	1,302,290	276	95.1
FRE-Bta-3	Bovine	France (56)	2013	Blood	1,293,571	303	94.2
FRE-Bta-4	Bovine	France (25)	2013	Blood	1,310,333	274	94.5
FRE-Bta-5	Bovine	France (45)	2016	Blood	1,373,659	237	95.1
FRE-Bta-6	Bovine	France (62)	2018	Endocervical swab	1,386,438	222	95.1
FRE-Bta-7	Bovine	France (62)	2018	Endocervical swab	1,294,521	233	94.2
BE-Bta-1	Bovine	Belgium	2018	Aborted fetus blood (blotting paper)	1,369,871	237	94.8
FRE-Hsa-1	Human	France (68)	2016	Blood	1,376,795	234	94.8
FRE-Hsa-2	Human	France (68)	2016	Blood	1,378,093	209	94.8
FRE-Hsa-3	Human	France (68)	2016	Blood	1,346,422	314	93.1
FRE-Hsa-4	Human	France (68)	2017	Blood	1,288,273	423	90.4

### 3.3 Genome comparison

**Fig. 1:**
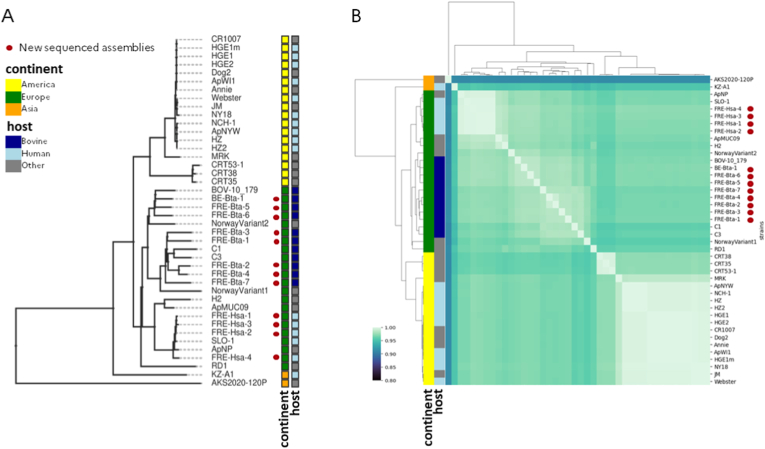
Comparative genetics analysis of *Anaplasma phagocytophilum*. **A** Kmer clustering of genomes was performed after mash analysis. The tree was constructed using the resulting pairwise genomic distances matrix between all genomes. **B** ANI clustering results. The newly sequenced assemblies from this study are indicated by red dots. The annotation includes the continent of origin and the host. For host classification, “Others” includes dogs, horses, rodents, sheep, roe deer, a marmot, and ticks. Detailed strain information is provided in Table 1.

We computed the pairwise genomic distances using the k-mer approach with mash on the 12 newly sequenced genome draft assemblies and the curated public database of 30 genomes to evaluate their overall genomic relatedness and position within the known diversity of the species. Clustering using the neighbor-joining (NJ) algorithm revealed two main clades corresponding to American and European isolates, respectively. Within the European cluster, an additional sub-clustering consistent with a differentiation of the European bovine and human samples was also observed (Fig. 1A).

An Average Nucleotide Identity (ANI) analysis was then performed to confirm the distances between the American and European assemblies, as well as between the European bovine and human assemblies. We observed the same division, supporting the presence of geographically structured lineages within *A. phagocytophilum*. Moreover, ANI enabled us to determine that all assemblies remained above the species-level threshold (≥ 95%), whereas the American and European clusters comparison exhibited lower ANI values, suggesting a lineage differentiation between them (Fig. 1B).

To further investigate the population structure of *A. phagocytophilum*, we conducted a genome-wide phylogenetic analysis based on core-SNPs using NY18 as the reference genome. To quantify genetic differentiation, pairwise single nucleotide polymorphism (SNP) distances were calculated and compared within and between continental groups. The mean pairwise SNP distance among the USA samples was 6582 SNPs, whereas the European assemblies displayed a higher within-group diversity, with a mean of 12,588 SNPs. In contrast, comparisons between the USA and European assemblies revealed a significantly greater mean genetic distance of 22,529 SNPs (Mann-Whitney U test, *P* < 0.001).

**Fig. 2:**
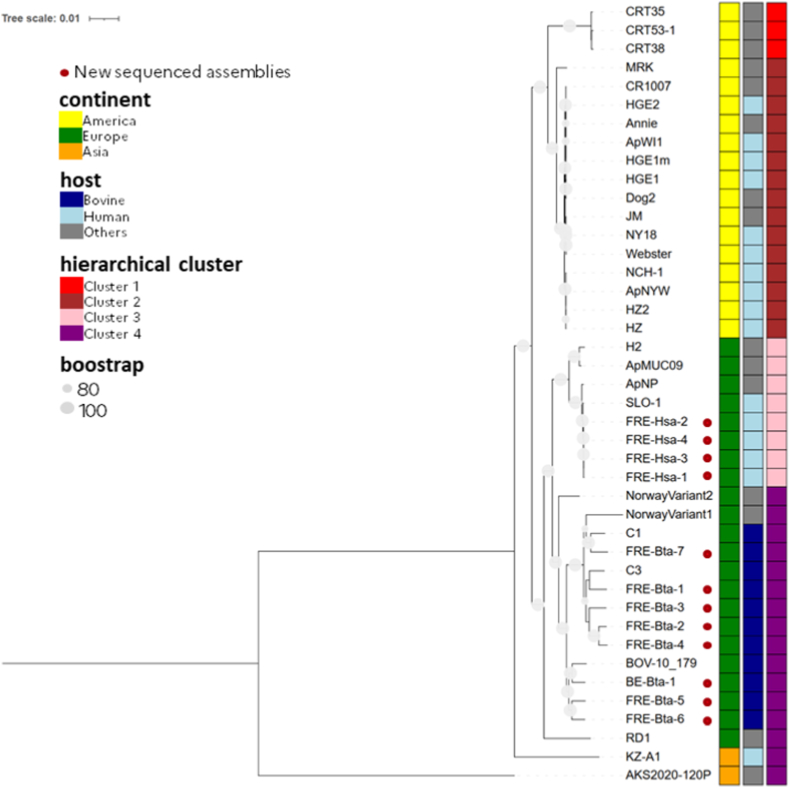
Phylogenetic analysis of *Anaplasma phagocytophilum*. The tree is annotated regarding isolates geographical origin, host and hierarchical Bayesian identified clusters. The newly sequenced assemblies from this study are indicated by red dots. Bootstrap support values are shown as grey circles, with circle size proportional to bootstrap support. The tree annotation includes the continent of origin, the host, and FastBAPS clustering results. For host classification, “Others” includes dogs, horses, rodents, sheep, roe deer, a marmot, and ticks. Detailed strain information is provided in Table 1.

The phylogenetic reconstruction revealed a clear separation between the samples from the USA and Europe, consistent with clustering patterns previously observed using Mash distance and ANI (Fig. 2). Bayesian hierarchical clustering identified four major genetic clusters. Clusters 1 and 2 comprised American assemblies, with Cluster 1 predominantly associated with tick-derived samples (Ap-V1 variant) and Cluster 2 corresponding to the zoonotic Ap-ha variant. The European human samples grouped into a single, well-defined cluster (e.g. Cluster 3) that also included two canine and one equine samples. Cluster 4 encompassed the remaining European assemblies together with Asian samples. Recombination analysis using ClonalFrameML revealed heterogeneous evolutionary dynamics among clusters. Clusters 1 and 2 showed low r/m values (0.41–0.75), indicating predominantly clonal evolution, whereas Clusters 3 and 4 exhibited higher recombination contributions with higher r/m values (2.9–5.0).

Pairwise *F*_ST_ (fixation index) analysis revealed pronounced genetic differentiation among population clusters, with *F*_ST_-values greater than 0.15 indicating high genetic differentiation according to commonly used population genetic thresholds (Wright, 1965). Significant divergence was observed between all USA and European clusters, indicating a strong geographical population structure. Within the USA isolates, the two clusters showed marked differences between samples derived from ticks *versus* humans, whereas the differences among European assemblies were less pronounced (Table 3). Altogether, these complementary analyses support the existence of two highly distinct lineages primarily structured by geography.

**Table 3:** *F*_ST_ comparison results between hierarchical clusters.

Comparison	Description	*F*_ST_	Population differences
Cluster 1 (*n* = 3) *vs* Cluster 2 (*n* = 16)	USA ticks *vs* USA human	0.67	Very high differentiation
Cluster 1 (*n* = 3) *vs* Cluster 3 (*n* = 9)	USA ticks *vs* European human	0.36	Very high differentiation
Cluster 1 (*n* = 3) *vs* Cluster 4 (*n* = 16)	USA ticks *vs* European others	0.16	High differentiation
Cluster 2 (*n* = 16) *vs* Cluster 3 (*n* = 9)	USA human *vs* European human	0.51	Very high differentiation
Cluster 2 (*n* = 16) *vs* Cluster 4 (*n* = 16)	USA human *vs* European others	0.23	High differentiation
Cluster 3 (*n* = 9) *vs* Cluster 4 (*n* = 16)	European human *vs* European others	0.08	Moderate differentiation

*Notes*: *F*_ST_ < 0.05 indicates very low genetic differentiation, *F*_ST_ of 0.05–0.15 – moderate differentiation, *F*_ST_ of 0.15–0.25 – high differentiation, and *F*_ST_ > 0.25 – very high differentiation) (Wright, 1965).

### 3.4 Pangenome comparison

**Fig. 3:**
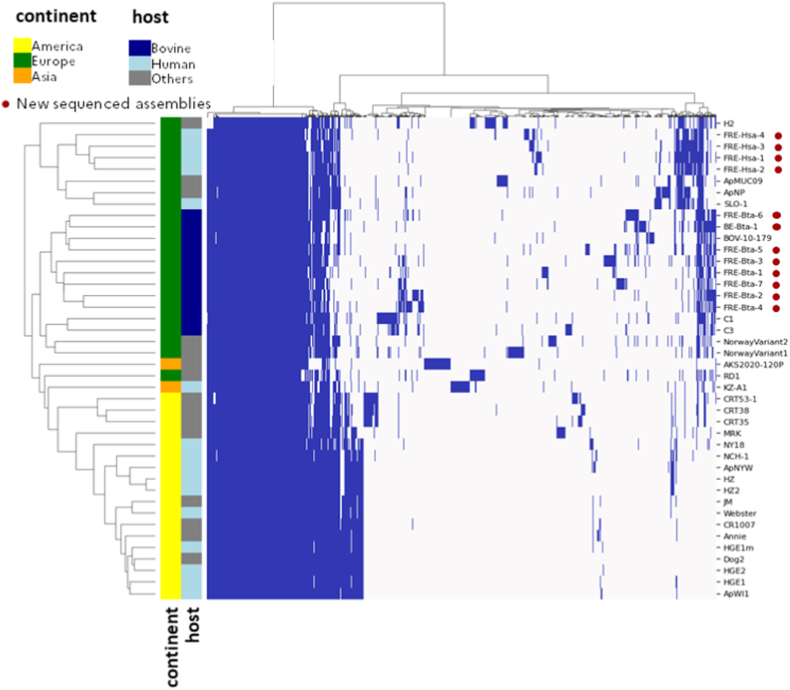
Pangenome of *Anaplasma phagocytophilum*: heatmap showing gene presence (*blue*) or absence (*white*) in each of the 42 *A. phagocytophilum* genomes. The newly sequenced assemblies from this study are indicated by red dots. The heatmap annotation includes the continent of origin and the host. For host classification, “Others” includes dogs, horses, rodents, sheep, roe deer, a marmot, and ticks. Detailed strain information is provided in Table 1.

The pangenome of *A. phagocytophilum* was inferred using PPanGGoliN at the limited scale of 42 genomes. A total of 3526 genes were identified through this analysis. The core genome consisted of 705 genes present in > 95% of samples (i.e. ≥ 40/42 genomes), which aligns closely with the findings of previous studies on *A. phagocytophilum* that reported similar restraint core sizes (e.g. core represents 20% of the pangenome). The accessory genome encompassed 2821 gene families, partitioned into a shell (moderately frequent genes) and cloud (rare genes). The shell included 560 genes present in 20–95% of samples (i.e. 9–39 assemblies), and the cloud comprised 2261 genes found in < 20% of samples (i.e. < 9 assemblies) (Fig. 3 and Table 4).

**Table 4:** Pangenome partition computed for all *A. phagocytophilum* isolates, and after grouping isolates regarding their geographical origin (Europe and USA).

	Core genes	Accessory genes
All isolates (*n* = 42)	705 (20%)	2821 (80%)
USA isolates (*n* = 18)	829 (54%)	714 (46%)
European isolates (*n* = 22)	619 (22%)	2199 (78%)

When inferring the pangenome per continent (Europe and North America), we found equivalent core genome sizes in the USA and European assemblies. However, we observed a significant smaller accessory gene size with only 714 accessory genes in the USA samples while 2199 accessory genes were found amongst the European assemblies, indicating a higher genetic heterogeneity within the European assemblies tested. This result was consistent with ANI and phylogeny results showing higher diversity in European assemblies. In contrast, the available USA assemblies were characterized by a larger core-genome proportion and a comparatively limited accessory genome, further supporting the clonal population structure observed in this lineage (Table 4).

### 3.5 Identification of potential zoonotic markers

To identify potential zoonotic markers of *A. phagocytophilum*, we performed GWAS using a gene presence-absence matrix derived from pangenome inference, comparing 15 human-derived *A. phagocytophilum* assemblies with other assemblies from various hosts. This approach revealed 10 candidate zoonotic markers, which were independent of the geographical origin of the strains. Functional annotation shows that these loci include two transcriptional regulators, two membrane-associated components, including a P44 outer membrane variant (e.g. *msp2* gene), and six genes of unknown function (Supplementary Table S2).

## 4 Discussion

Despite the significant impact of granulocytic anaplasmosis on human health in the USA and on ruminant herds in Europe, only 34 assembled *A. phagocytophilum* genomes were publicly available prior to this study. This limited availability reflects the difficulty of sequencing this obligate intracellular bacterium, which resides within host neutrophils. Previous studies have used diverse sequencing strategies, including short-read (Illumina®) and long-read (PacBio®, ONT®) approaches, as well as targeted and shotgun methods. The variety of sequencing methods can introduce differences in assembly and variable completeness, potentially affecting comparative genomic analyses. By screening for contamination and assessing completeness with BUSCO, we filtered out four assemblies that were incomplete, fragmented, and/or contained host-derived sequences. Removing host contigs from assemblies further improved the quality of the available *A. phagocytophilum* genomic database and improved downstream analyses, too.

Moreover, the majority of publicly available genomes are derived primarily from USA samples, particularly human ones. In contrast, only one human sample from Slovenia has been sequenced in 2025 (Lesiczka et al., 2025). Expanding the representation of European genomes is therefore essential to better characterize the genetic diversity of *A. phagocytophilum*. However, obtaining new genome draft assemblies from clinical samples remains challenging, as illustrated by the Slovenian sample, which required fastidious culture in HL-60 cells prior to sequencing. In this study, we describe 12 new European *A. phagocytophilum* genome draft assemblies from bovine and human hosts, more than doubling the number of available European *A. phagocytophilum* genomes. These genome draft assemblies were obtained directly from blood or endocervical swab samples using an improved Illumina® targeted sequence capture, without the need for bacterial culture. This is particularly invaluable for ruminant samples, for which the only option is culturing on tick cell lines, a tedious and slow process with variable success. The capture probes used here were optimized to enhance the recovery of *A. phagocytophilum* DNA, even from samples with low abundance. This approach allows the use of field-collected samples and overcomes limitations of earlier sequencing attempts, which produced incomplete assemblies with multiple scaffolds and host contamination (Dugat et al., 2014, 2016).

Despite the limited number and diversity of European and American *A. phagocytophilum* genomes, we were able to perform a comprehensive comparative analysis of genomes originating in the USA and Europe using this expanded cohort. Our analysis of 42 genomes, including 30 curated public genomes and 12 newly sequenced European genome draft assemblies, identified a core-genome (e.g. genes found in > 95% of isolates) of approximately 20% of total pangenome. Only one pangenome study has been reported previously (Crosby et al., 2022), which, based on 28 assemblies, identified a small core-genome of 501 genes (11% of the total pangenome) and 4060 accessory genes. These differences in core genome size can be explained by two factors. First, the increase in the conserved portion of the genome is likely due to the increased number of European samples. Secondly, we detected contamination in some publicly available genomes, which may have led to an overestimation of the proportion of accessory genes in previous studies by introducing non-bacterial genes. The core genome identified in this study remains relatively small and could reflect the high phylogenetic diversity observed among European and USA isolates, as well as between bovine- and human-associated isolates. It may indeed be influenced by distinct adaptation processes, where different ecological contexts may contribute to gene content variation. Differences in host range and tick vectors across geographical locations or host species could therefore play a role in shaping genome diversity, although further sequencing is required to confirm this hypothesis and to rule out potential biases due to technical factors. In any case, these results suggest that the accessory genome could contribute substantially to *A. phagocytophilum* evolution.

A recent comparison of 13 assemblies (Lesiczka et al., 2025) confirmed a clear distinction between the USA and European *A. phagocytophilum* genomes and highlighted the close relationship of the Slovenian human isolate with dog isolates. Phylogenetic analysis revealed four main clusters separating the USA and European genomes, with additional sub-clustering within the European clade reflecting distinct origins of bovine and human samples. ANI analysis confirmed the genetic distances between the USA and European genomes, as well as between bovine and European human ones. Together, these analyses indicate the existence of two highly distinct lineages structured primarily by geographical origin. This confirms what was already described by several authors using single-locus analysis (*ankA*, *groEL*) and multilocus sequence typing (Rar et al., 2021). It should be noted that, despite their limited number, the USA isolates originate from different, far-apart states (Wisconsin/Minnesota, New York, Massachusetts and California). Yet, they appear to be highly clonal, which is consistent with the limited diversity observed in the core and accessory genomes. By contrast, European isolates display greater diversity, likely reflecting the presence of multiple host species and distinct epidemiological cycles in Europe. This difference in diversity may also explain the more moderate genetic differentiation observed within European samples compared with the strong separation between the USA and European populations. Nevertheless, a clear genetic distinction remains between bovine and European human assemblies, strongly suggesting the existence of partially host-associated lineages within the European population, in accordance with previous studies (Jahfari et al., 2014; Jaarsma et al., 2019; Rar et al., 2021; Lesiczka et al., 2025).

A comparative genomic study suggested using the *drhm* gene to distinguish between zoonotic and non-zoonotic isolates of *A. phagocytophilum* (Al-Khedery and Barbet, 2014). However, a second study concluded that the absence of this gene was not an indicator of pathogenicity for humans, since all European isolates of the agent responsible for human granulocytic anaplasmosis are *drhm* positive (Langenwalder et al., 2020). By comparing human-derived *A. phagocytophilum* genomes and those obtained from other hosts, we were able to identify 10 potential new zoonotic markers of *A. phagocytophilum,* including a variant in the *msp2/p44* repertoire. Interestingly, Barbet et al. (2013) previously identified this last marker, which is responsible for host immunity escape through recombination between a single expression site that encodes a major surface antigen and more than 100 functional pseudogenes. All these markers represent promising targets for tracing bacterial reservoirs, refining our understanding of host range and zoonotic transmission of the bacterium, and improving tick- and host-based surveillance strategies.

However, our study remains limited by the relatively small number of available assemblies and the under-representation of certain host species, which constrains the resolution of both pangenome and host-specificity analyses. Fragmentation of the resulting draft assemblies was expected, given that we used a targeted capture-based short-read sequencing approach. Nevertheless, fragmentation is unlikely to have materially altered phylogenetic inference or ANI-based comparisons, as these are largely driven by shared orthologous genomic regions. The genome completeness of both the publicly available references and those generated in this study ranged from approximately 85% to over 95%, which is sufficient for phylogenetic inference and ANI analyses (Jain et al., 2018). However, this level of completeness may still affect pangenome analyses by leading to the absence of some coding sequences (CDS) and may limit the identification of potential zoonotic markers and underestimate core-genome size. New sequencing approaches, combining short-read and Oxford Nanopore long-read technologies, have been successfully applied to obtain high-quality whole genomes of related *Anaplasmataceae* species, including *Ehrlichia canis* (Neave et al., 2022) and “*Candidatus* Neoehrlichia mikurensis” (Azagi et al., 2022). Applying similar hybrid approaches to *A. phagocytophilum* could improve the completeness of publicly available references and allow for continuing to increase the number of genomes available.

## 5 Conclusion

In conclusion, our results confirm a significant divergence between European and USA strains, with European genomes exhibiting greater genetic diversity than USA ones. Hence, capture-based short-read sequencing applied to samples from naturally infected humans and ruminants can increase the representation of European genomes, particularly human isolates, and refine the population structure of *A. phagocytophilum* beyond patterns previously inferred from limited gene-based approaches. Our approach also provided a framework for identifying potential genomic markers that could help define lineages associated with zoonotic transmission and improve the characterization of epidemiological cycles.

## Ethical approval

Studies on human blood samples was approved by the Ethics Committee of Strasbourg University (No CE-2020-175). The domestic animals used in this study met the definition of “farm animals”, which are not covered by French regulations (Decree n° 2013–118 implemented the 1st February 2013 issued by the French Ministry of Agriculture, Agri-Food, and Food Sovereignty). The animal owners’ consent was obtained prior to blood collection.

## CRediT authorship contribution statement

**Clotilde Rouxel:** Methodology, Investigation, Writing - original draft, Writing - review & editing. **Pierre Lucien Deshuillers:** Conceptualization, Writing - original draft, Writing - review & editing. **Meryl Vila-Nova:** Methodology, Writing - review & editing. **Deborah Merda:** Methodology, Writing - review & editing. **Pierre Boyer:** Resources, Writing - review & editing. **Benoit Jaulhac:** Resources, Writing - review & editing. **Claude Saegerman:** Resources, Writing - review & editing. **Laurent Delooz:** Resources, Writing - review & editing. **Grégoire Kuntz:** Resources, Writing - review & editing. **Henri-Jean Boulouis:** Conceptualization, Resources, Writing - review & editing, Funding acquisition. **Nadia Haddad:** Conceptualization, Resources, Writing - original draft, Writing - review & editing, Funding acquisition. **Anne-Claire Lagrée:** Conceptualization, Resources, Methodology, Investigation, Writing - original draft, Writing - review & editing, Supervision, Funding acquisition. **Virginie Chesnais:** Conceptualization, Methodology, Formal analysis, Investigation, Writing - original draft, Writing - review & editing, Visualization, Supervision.

## Funding

This work was supported by the French Government’s Investment for the Future program as a Laboratory of Excellence in “Integrative Biology of Emerging Infectious Diseases” (grant No. ANR-10-LABEX-62-IBEID). The Animal Health Department of INRAe supported specifically the sequencing of bovine samples.

## Declaration of competing interests

The authors declare that they have no known competing financial interests or personal relationships that could have appeared to influence the work reported in this paper.

## Data Availability

The data supporting the conclusions of this article are included within the article and its supplementary files. The 12 genome sequences of *A. phagocytophilum* were submitted to GenBank under Bioproject PRJNA1456935. The 30 curated genomes are available through Figshare (https://doi.org/10.6084/m9.figshare.32083359). The supplementary file contains the Ct values of *A. phagocytophilum* DNA in bovine and human samples, the description of the 30 curated public genomes, and the detailed GWAS results.
