# Supplementary material for: Exploration of geographical population structure of Anaplasma phagocytophilum: Insights from 12 newly sequenced European human and bovine genome assemblies
Source: Curr Res Parasitol Vector Borne Dis. 2026 May 27;9:100393. doi: 10.1016/j.crpvbd.2026.100393 (PMC13251642; doi:10.1016/j.crpvbd.2026.100393)
Supplement: Multimedia component 1 [file mmc1.pdf]

Supplementary file 1

Supplementary Table S1. Detection of *A. phagocytophilum* DNA in bovine and human samples by *msp2* qPCR.

Isolate ID	Ct
**FRE-Bta-1**	26.85
**FRE-Bta-2**	26.22
**FRE-Bta-3**	27.25
**FRE-Bta-4**	24.81
**FRE-Bta-5**	23.67
**FRE-Bta-6**	21.83
**FRE-Bta-7**	22.38
**BE-Bta-1**	24.79
**FRE-Hsa-1**	25.26
**FRE-Hsa-2**	22.34
**FRE-Hsa-3**	21.20
**FRE-Hsa-4**	21.75

Supplementary Table S2. GWAS results highlighting genes associated with zoonotic potential of *A. phagocytophilum*. Frequency of genes occurrence in human and non-human strains is displayed. af: Proportion of individuals in the population carrying the gene; lrt-pvalue: Likelihood Ratio Test p-value, corrected; beta: Estimated effect of the allele on the trait.

Gene identification	Description	Frequency in human isolates	Frequency in non-human isolates	af	lrt-pvalue	beta
CDS_0982	P44 outermembrane	69% (11/16)	27% (7/26)	0.450	0.0147	1.79
CDS_0649	Efflux RND transporter periplasmic adaptor subunit	113% (2/16)	65% (17/26)	0.475	0.0031	-4.90
CDS_0697	Transcriptional regulator	81% (13/16)	35% (9/26)	0.550	0.0133	2.09
CDS_0191	Transcriptional regulator	81% (13/16)	31% (8/26)	0.525	0.0085	3.02
CDS_0657	Hypothetical protein	56% (9/16)	12% (3/26)	0.300	0.0044	2.24
CDS_0366	Hypothetical protein	31% (5/16)	23% (6/26)	0.275	0.0074	3.69
CDS_0654	Hypothetical protein	69% (11/16)	73% (19/26)	0.725	0.0156	-2.94
CDS_1387	Hypothetical protein	63% (10/16)	23% (6/26)	0.400	0.0006	3.08
CDS_1127	Hypothetical protein	75% (12/16)	42% (11/26)	0.575	0.0136	1.94
CDS_0334	Hypothetical protein	31% (5/16)	23% (6/26)	0.275	0.0047	4.03

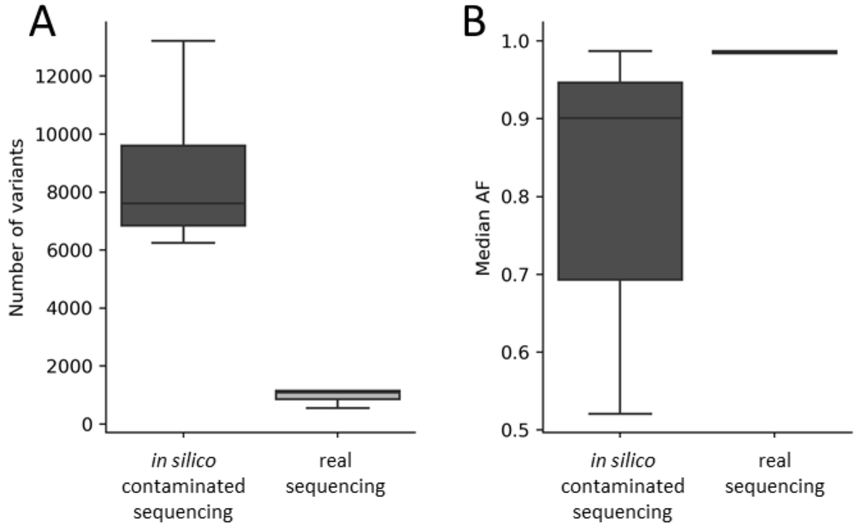

Supplementary Figure S1. Detection of subclonal mutations in real sequencing data and *in silico* artificially mixed-infected samples with multiple Aph strains. **A** Distribution of the number of identified variants per sample. **B** Distribution of allele frequencies for the detected mutations.
